# Real-world use of efgartigimod in acetylcholine receptor antibody–positive generalized myasthenia gravis: experience from two centers in Greece and Cyprus

**DOI:** 10.3389/fneur.2026.1755374

**Published:** 2026-01-21

**Authors:** Tasos Tsokkos, Eleni Strataki, Dimitra Tzavella, Eleni Zamba-Papanicolaou, Kleopas A. Kleopa, Vasiliki Zouvelou

**Affiliations:** 1 Center for Neuromuscular Disorders, The Cyprus Institute of Neurology and Genetics, Nicosia, Cyprus; 2 1st Department of Neurology, Eginition Hospital, National and Kapodistrian University of Athens, Athens, Greece; 3 Department of Neuroscience, The Cyprus Institute of Neurology and Genetics, Nicosia, Cyprus

**Keywords:** AChR-positive MG, corticosteroid tapering, efgartigimod, FcRn inhibition, generalized myasthenia gravis, longitudinal management, MG-ADL, real-world evidence

## Abstract

**Introduction:**

Efgartigimod, an FcRn antagonist that reduces circulating immunoglobulin G (IgG), is approved for the treatment of acetylcholine receptor (AChR) antibody–positive generalized myasthenia gravis (gMG). While its efficacy has been demonstrated in randomized clinical trials, real-world data describing longitudinal use and management of efgartigimod in routine clinical practice are still emerging.

**Methods:**

We conducted a retrospective observational study across two neuromuscular referral centers, including adults with AChR antibody–positive gMG treated with intravenous efgartigimod between April 2023 and August 2025. Treatment was initiated across a range of real-world clinical scenarios, including refractory disease, non-crisis symptom relapse, corticosteroid tapering, maintenance of minimal-symptom expression, and bridging before thymectomy or initiation of conventional immunosuppression. Retreatment intervals were individualized based on clinical course. Clinical outcomes, corticosteroid use, serological markers, adjunctive therapies, and safety events were analyzed descriptively.

**Results:**

Thirteen patients received a total of 52 efgartigimod treatment cycles. Most patients experienced repeated improvement in MG-Activities of Daily Living (MG-ADL) during treatment cycles, although clinical status varied over time. Corticosteroid doses were reduced in the majority of patients during follow-up. Episodes of clinically significant worsening occurred outside the setting of myasthenic crisis and were managed with established rescue therapies, after which efgartigimod could often be resumed. Treatment was generally well tolerated.

**Conclusion:**

This study describes how efgartigimod is integrated into routine clinical practice as part of longitudinal disease management for AChR antibody–positive gMG. Real-world use encompassed diverse clinical scenarios and required individualized retreatment decisions and ongoing reassessment. These findings highlight the role of FcRn blockade as a flexible therapeutic option that complements established therapy in everyday care.

## Introduction

Myasthenia gravis (MG) is an autoimmune disorder of the neuromuscular junction characterized by fluctuating skeletal muscle weakness due to impaired synaptic transmission. In most patients with generalized MG (gMG), pathogenic IgG1 and IgG3 antibodies target the acetylcholine receptor (AChR), leading to complement-mediated receptor loss and end-plate damage. Other subgroups include patients with antibodies against muscle-specific kinase (MuSK), low-density lipoprotein receptor–related protein 4 (LRP4), or seronegative disease (1, 2).

Standard treatment combines symptomatic acetylcholinesterase inhibition with corticosteroids and other immunosuppressive agents, while intravenous immunoglobulin (IVIG) or plasma exchange (PLEX) are reserved for acute exacerbations (3). Despite their effectiveness, many patients experience incomplete disease control, treatment dependence, or cumulative toxicity, highlighting the need for targeted therapies with improved tolerability (4, 5).

Efgartigimod, an engineered human IgG1 Fc fragment with high affinity for the neonatal Fc receptor (FcRn), exploits FcRn-mediated IgG recycling to selectively reduce total IgG and disease-specific autoantibody levels through accelerated IgG catabolism, without global immunosuppression (6–9). In the phase 3 ADAPT trial and its open-label extension ADAPT+, efgartigimod demonstrated significant clinical benefit and favorable tolerability in patients with AChR antibody–positive gMG (10, 11). Subsequent real-world data from Asia and Europe have further substantiated its clinical benefit, rapid onset of action, steroid-sparing potential, and safety in routine practice (12–15).

In this study, we describe the real-world use of efgartigimod in patients with AChR antibody–positive gMG across different clinical scenarios and treatment strategies encountered in routine care at two neuromuscular referral centers in Greece and Cyprus. The descriptive approach adopted allows depiction of within-patient response patterns over time and supports a pragmatic understanding of how FcRn blockade is applied in real-world treatment contexts and clinician decision-making outside protocolized trial settings.

## Methods

### Study design and setting

We conducted a retrospective, observational study of patients with gMG treated with efgartigimod at two neuromuscular centers: the Cyprus Institute of Neurology and Genetics (Nicosia, Cyprus) and First Department of Neurology, University of Athens. The study period spanned from April 2023 to August 2025.

### Eligibility criteria

Eligible patients were adults (≥ 18 years) with a confirmed diagnosis of generalized myasthenia gravis according to established criteria. Only acetylcholine receptor (AChR) antibody–positive cases, consistent with the approved indication for efgartigimod by the European Medicines Agency, used as add-on therapy to standard care, were included (16). While MG-Activities of Daily Living (MG-ADL) thresholds were applied as inclusion criteria in pivotal clinical trials, the approved indication does not include a specified minimum MG-ADL score. Patients with isolated ocular MG, MuSK-positive MG, or seronegative MG were not treated with efgartigimod at these centers. Patients were eligible for efficacy analyses if they had at least one treatment cycle with available MG-ADL assessments both before and after efgartigimod administration.

### Treatment

Efgartigimod was administered intravenously at a dose of 10 mg/kg once weekly for four consecutive weeks (one treatment cycle), in accordance with the approved regimen. In routine clinical practice, the timing of subsequent cycles was individualized and determined by the treating clinician based on the patient’s clinical status and practical considerations, including intercurrent infections or comorbidities. Accordingly, additional cycles were administered at variable intervals as part of observational, real-world care rather than according to a prespecified or protocolized retreatment schedule.

### Data collection

Data were extracted from medical records, including: (a) Demographics: sex, age at efgartigimod initiation, and disease duration, (b) Clinical severity: Myasthenia Gravis Foundation of America (MGFA) class at baseline and Myasthenia Gravis Activities of Daily Living (MG-ADL) scores immediately before and after each cycle. (c) Immunological markers: baseline AChR antibody positivity in all patients; follow-up AChR titers (measured by radioimmunoassay, RIA) and total IgG levels when available. (d) Corticosteroid therapy: daily prednisolone-equivalent dose before and after each cycle and at the most recent follow-up. (e) Concomitant immunosuppressive therapy: use of oral agents such as azathioprine or mycophenolate mofetil, including whether treatment was continued, discontinued or initiated. (f) Treatment exposure: number of cycles, interval between cycles, total duration of follow-up, and reasons for discontinuation, and (g) Safety: infections, infusion-related reactions, and other adverse events occurring during treatment.

### Analysis populations

The analysis included two predefined populations to distinguish efficacy evaluation from overall treatment exposure. (a) Primary Efficacy Set (PES): all patients and treatment cycles with baseline (pre-cycle) MG-ADL ≥ 2, to minimize floor effects and capture clinically meaningful change. (b) Full Analysis Set (FAS): all 13 treated patients (and their cycles) with any available MG-ADL data, irrespective of baseline score. Cycles starting at MG-ADL = 0 or 1 were excluded from the primary efficacy analysis because a ≥ 2-point improvement cannot occur from that baseline. These were analyzed separately as maintenance, corticosteroid-sparing, or bridging treatments, reflecting preservation of minimal-symptom status rather than absence of efficacy.

### Outcomes

Primary outcome was considered the change in MG-ADL score from pre- to post-cycle within the PES. Secondary outcomes were: (a) Responder rate (≥ 2-point MG-ADL improvement from pre- to post-cycle) analogous to the responder definition used in ADAPT; (b) achievement of minimal-symptom expression (MSE), defined as MG-ADL 0–1, as defined in ADAPT+; (c) corticosteroid-sparing effect, assessed as (i) per-cycle change in prednisone-equivalent dose and (ii) overall change from baseline to the latest follow-up; (d) changes in total IgG and AChR-antibody titers, where available; and (e) safety outcomes: infections, infusion reactions, and treatment discontinuations.

### Statistical analysis

Continuous variables were summarized as mean ± SD or median (IQR), and categorical variables as counts (%). Per-cycle MG-ADL changes were primarily evaluated descriptively using medians and interquartile ranges, while overall within-patient change from baseline to the latest follow-up in the PES was additionally assessed using paired *t*-test. Changes in total IgG and AChR-antibody levels before and after each cycle were analyzed using the Wilcoxon signed-rank test for paired comparisons. Associations between the magnitude of total IgG or AChR-antibody reduction and MG-ADL improvement were examined using Pearson’s (r) and Spearman’s (*ρ*) correlation coefficients. Responder rates (≥ 2-point improvement) and achievement of minimal-symptom expression (MG-ADL 0–1) were expressed as percentages. Given the small sample size, analyses were primarily descriptive, with inferential tests performed on an exploratory basis.

## Results

### Patient disposition and analysis populations

**Figure 1:**
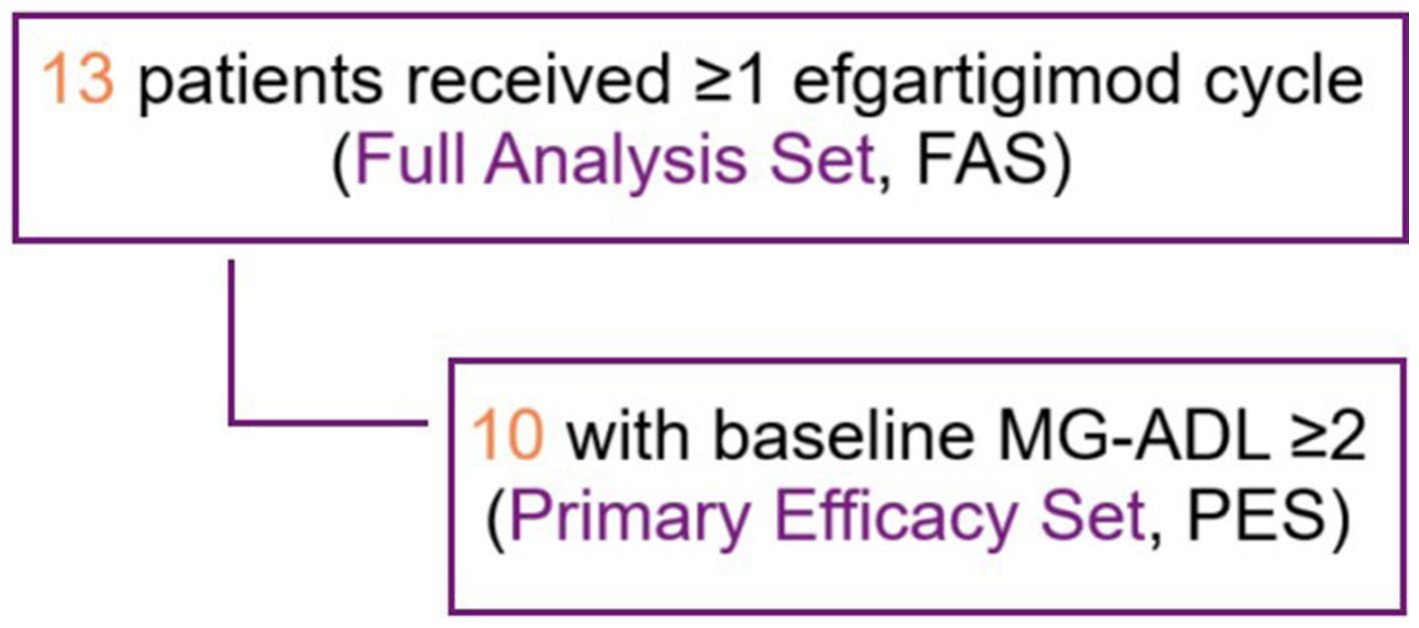
Flow diagram illustrating patient disposition across analysis sets. Of 13 patients who received at least one efgartigimod cycle (full analysis set, FAS), 10 had baseline MG-ADL ≥ 2 and were included in the primary efficacy set (PES).

A total of 13 patients with AChR-antibody–positive gMG received at least one efgartigimod treatment cycle between April 2023 and August 2025. All were included in the Full Analysis Set (FAS). The Primary Efficacy Set (PES) comprised 10 patients who had at least 1 cycle with baseline MG-ADL ≥ 2 (Figure 1). Three patients were treated exclusively during periods of minimal-symptom expression (baseline MG-ADL 0–1), such as maintenance or bridging treatment. In total, the cohort contributed 52 treatment cycles.

### Baseline characteristics and treatment exposure

While most patients followed the standard regimen of four weekly infusions per cycle, one received a modified intermittent schedule consisting of two consecutive weekly infusions followed by a one-week pause—occasionally extended to 2 weeks during 1 cycle. This individualized, clinician-driven approach aimed to maintain clinical stability and convenience while minimizing treatment burden. The interval between cycles was defined as the time from the start date of one efgartigimod cycle to the start date of the subsequent cycle. Across the cohort, the median start-to-start interval was 9 weeks (IQR 7–11; range 6–29). Two patients permanently discontinued efgartigimod during follow-up: one due to gradual attenuation of treatment effect over successive treatment cycles and another one following a new diagnosis of malignancy, deemed unrelated to efgartigimod, with treatment discontinued to initiate oncologic management. Baseline demographics and clinical features are summarized in Table 1.

**Table 1:** Baseline demographic and clinical characteristics of the study cohort.

Characteristic	Value
Number of patients	13
Age at initiation, years, median (IQR), range	47 (36–67), 26–88
Sex, *n* (%)	Females 8 (62), Males 5 (38)
Disease duration, years, median (IQR), range	6.4 (1.2–10.6), 0.2–31.5
Number of cycles received, median (range)	4 (1–8)
Cycle interval (start-to-start), weeks, median (IQR), range	9 (7–11), 6–29

Summary of baseline characteristics for the 13 patients included in the study. Values are presented as number (%), median (interquartile range [IQR]), and range, as appropriate.

At baseline, disease severity according to the MGFA classification ranged from class IIIa to IVb. Most patients (9/13) were classified as MGFA III (six IIIb and three IIIa), while one patient had class IVb disease. Three patients had MSE (MG-ADL 0–1), reflecting minimal disease activity at baseline; therefore, MGFA classification was not applied.

### Clinical indications and treatment rationale

Efgartigimod was most commonly initiated (in eight patients) in the setting of incomplete disease control despite corticosteroids and conventional immunosuppressive therapies, and in some cases despite maintenance treatment with IVIG, or following non–life-threatening relapse with symptom recurrence. One patient received efgartigimod after secondary loss of response to ravulizumab. In two immunosuppressant-naïve patients with long-standing gMG and residual limb weakness accompanied by intermittent symptomatic fluctuations, efgartigimod was introduced as add-on therapy to pyridostigmine.

Among the three patients treated during periods of MSE, distinct clinical contexts were observed. In one young female patient, efgartigimod was administered in the peri-operative period prior to thymectomy. At that time, she was receiving high-dose oral prednisolone (55 mg/day), and efgartigimod was introduced to facilitate corticosteroid tapering to 20 mg/day while maintaining clinical remission, with the aim of reducing the risk of peri-operative complications associated with prolonged high-dose corticosteroid exposure. The remaining two patients had late-onset gMG and received efgartigimod as bridging therapy until the clinical effect of a non-steroidal immunosuppressive agent was established. One of these patients had experienced two severe, unprovoked exacerbations within the preceding 6 months, necessitating rescue therapy with IVIG and PLEX, respectively. In the other patient, long-term oral corticosteroid therapy was contraindicated because of severe osteoporosis; therefore, efgartigimod was selected as a temporary treatment option while awaiting the onset of action of conventional immunosuppression. In three patients with a history of extrathymic malignancy (two with refractory disease and one treated during MSE), efgartigimod was preferentially selected to avoid non-selective immunosuppression and to minimize the theoretical risk of cancer recurrence. Individual patient characteristics and treatment details are summarized in Supplementary Table S1.

### MG-ADL outcomes

**Figure 2:**
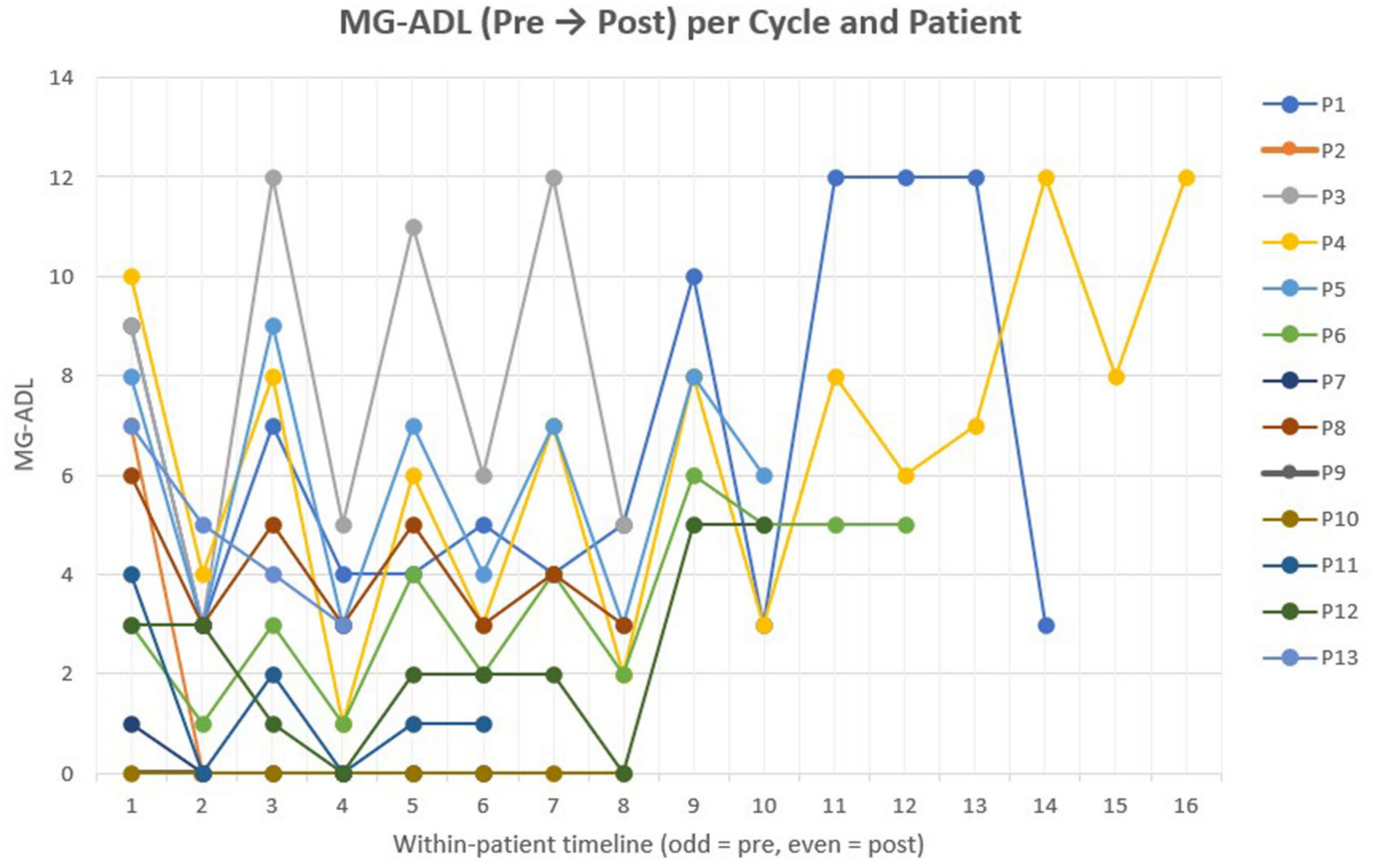
Individual MG-ADL trajectories across treatment cycles. Line plot showing MG-ADL scores before and after each efgartigimod treatment cycle in 13 patients. Each line represents one patient, illustrating within-patient variation and longitudinal trends in clinical response. Odd-numbered points indicate pre-cycle assessments, and even-numbered points post-cycle measurements.

At baseline, the median MG-ADL score across all patients (FAS; *n* = 13) was 6 (IQR 2–9; range 0–10). Within the PES (*n* = 10), the median baseline score was 7 (IQR 4–9). After excluding cycles initiated at MG-ADL 0–1, a total of 42 efficacy-evaluable cycles were analyzed. The median per-cycle MG-ADL change was −2 points (IQR − 4 to −2). A ≥ 2-point improvement was observed in 31 cycles (74%), stability (± 1 point) in 8 (19%), and worsening in 2 (5%), both from the same patient who exhibited late clinical fluctuation after repeated cycles. Across the treatment period, all PES patients achieved at least one ≥ 2-point MG-ADL improvement, and half (5/10) reached MSE during at least 1 cycle. From baseline to the latest follow-up, MG-ADL scores among PES patients decreased significantly, with a median overall change of −3 points (IQR − 5 to 0) and a mean reduction from 6.7 ± 2.4 to 4.3 ± 3.2 (*t* (9) = 2.58, *p* = 0.030). At the latest assessment, seven patients (70%) maintained lower MG-ADL scores compared with baseline, while three (30%) showed an increase of +2 points. Figure 2 illustrates individual MG-ADL trajectories across treatment cycles.

### Corticosteroid outcomes

**Figure 3:**
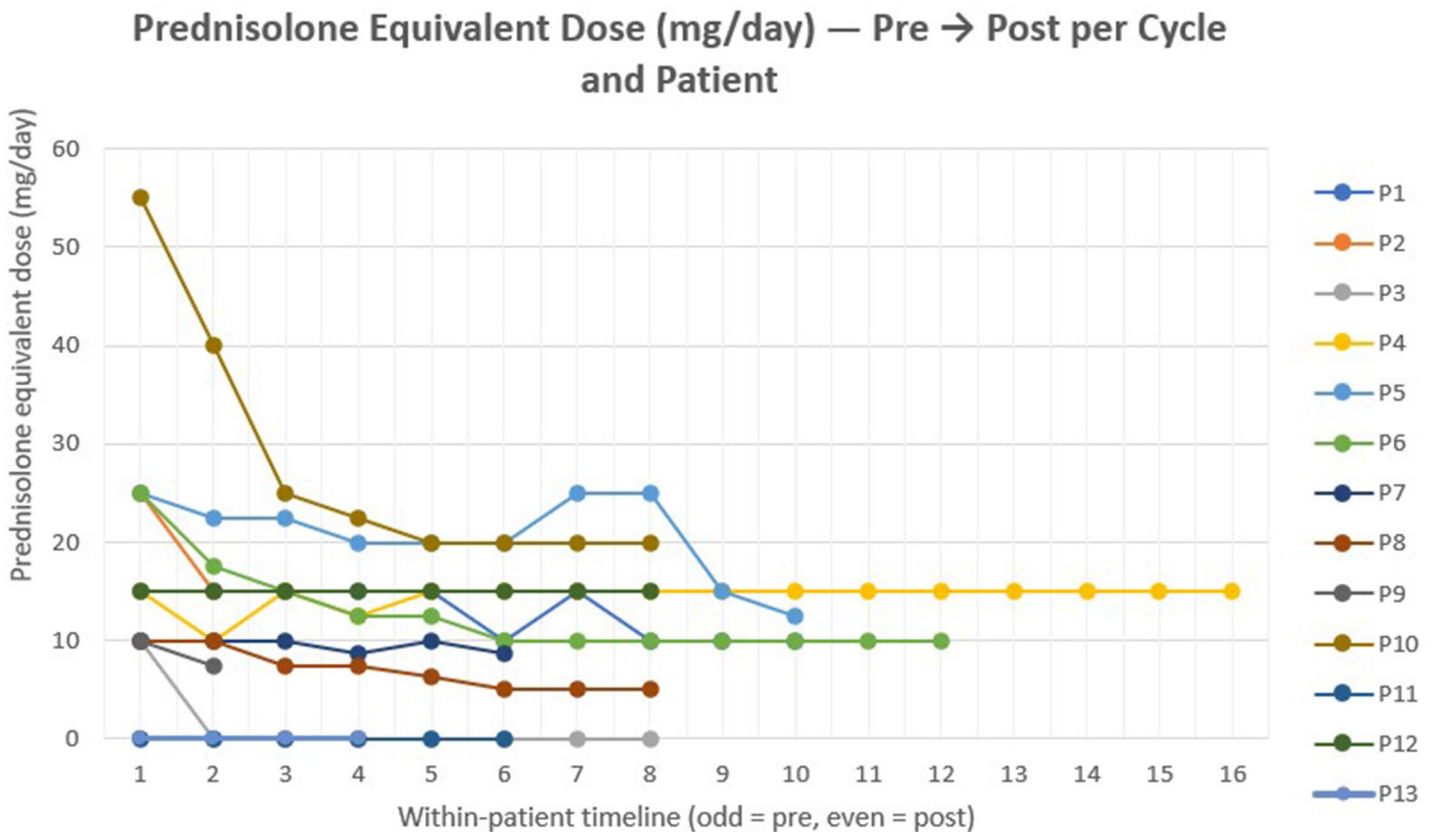
Individual corticosteroid trajectories. Line plot showing prednisone-equivalent dose (mg/day) the course of steroid dose changes for each patient in relation to the efgartigimod treatment cycles. For alternate-day regimens, the mean daily equivalent dose was calculated (e.g., 20/10 mg → 15 mg/day).

At baseline, 11 of 13 patients (85%) were receiving oral corticosteroids, with a median prednisolone-equivalent dose of 15 mg/day (IQR 10–22.5 mg). During follow-up, 9 of these 11 patients (82%) achieved a dose reduction, including one who successfully discontinued corticosteroids. Among those who tapered, the median decrease was 10 mg/day (IQR 5–15 mg), corresponding to an approximate 50% reduction from baseline doses. The final median daily dose across all steroid-treated patients was 10 mg (IQR 8.8–17.5 mg), while two patients (18%) were maintained on stable regimens without dose change. Figure 3 illustrates individual corticosteroid trajectories (prednisolone-equivalent doses) across treatment cycles.

### Oral immunosuppressive therapies

At baseline, 4 of 13 patients (31%) were receiving concomitant oral immunosuppressive therapy in addition to corticosteroids—one on azathioprine (AZA) and three on mycophenolate mofetil (MMF). During efgartigimod treatment, doses remained stable in two patients, while azathioprine and mycophenolate were discontinued in two others because of lymphopenia and were not reinitiated. In one patient, efgartigimod was introduced to bridge the delayed onset of action of a newly started MMF.

### Serological outcomes (total IgG and AChR antibodies)

Across all paired cycles (FAS, *n* = 34), total IgG levels decreased significantly after each efgartigimod cycle (median reduction 56.7%, IQR 44.5–65.3%; Wilcoxon *p* < 0.001), consistent with the expected pharmacodynamic effect of FcRn blockade. Within the PES (baseline MG-ADL ≥ 2; *n* = 25), the median per-cycle IgG reduction was 54.4% (IQR 39.2–65.9%; *p* < 0.001). The correlation between percent IgG reduction and MG-ADL improvement was weak and not statistically significant (Pearson *r* = 0.20, *p* = 0.34; Spearman *ρ* = 0.29, *p* = 0.16).

**Figure 4:**
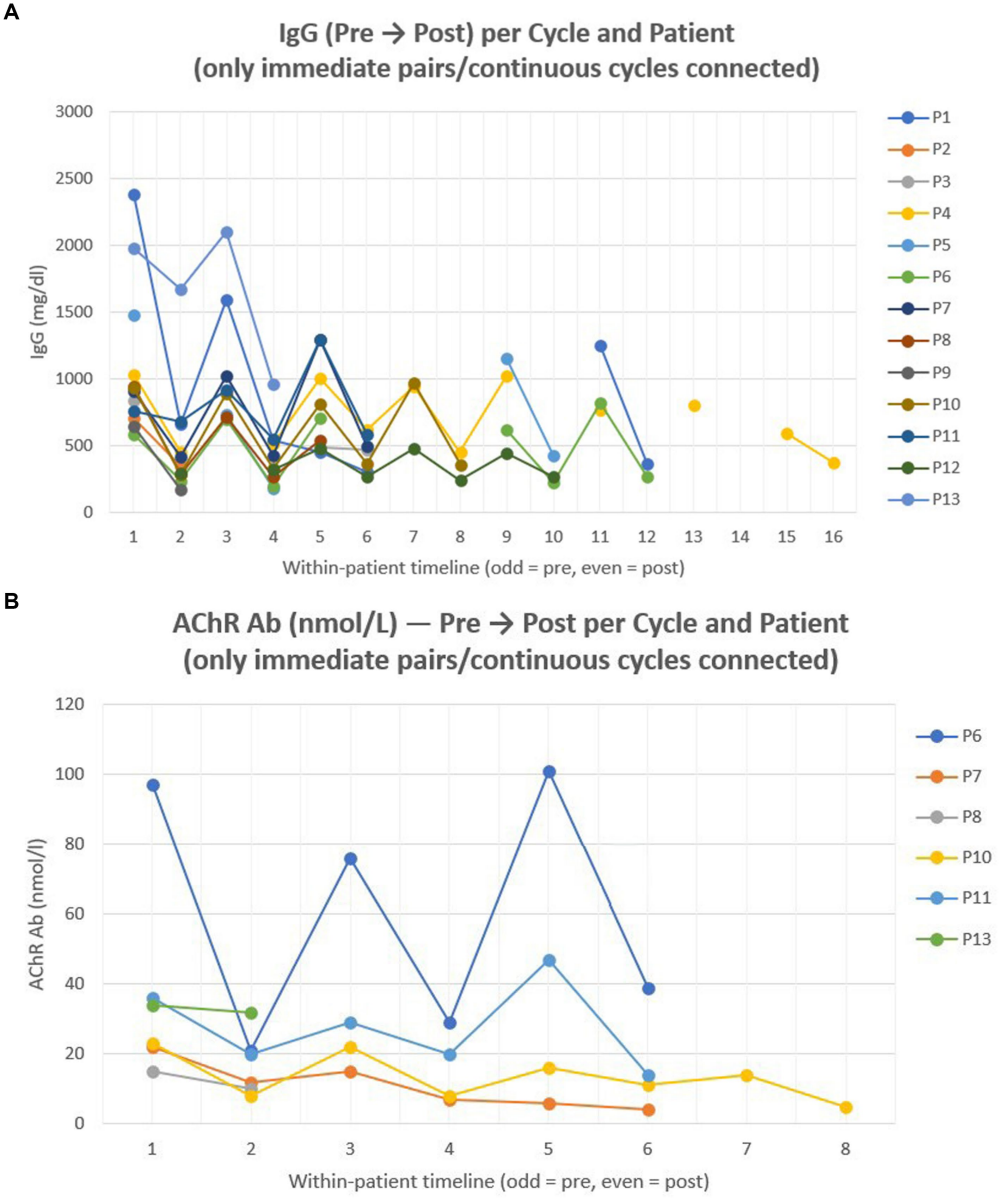
Individual IgG and AChR antibody trajectories across treatment cycles. Line plot showing total IgG **(A)** and acetylcholine receptor (AChR) antibody titres **(B)** before and after efgartigimod treatment cycles in patients with available paired measurements. Each line represents one patient, demonstrating within-patient variation and longitudinal changes in IgG and AChR antibody levels during therapy. Odd-numbered points correspond to pre-cycle assessments, and even-numbered points to post-cycle measurements.

Paired acetylcholine receptor (AChR) antibody titers were available in 15 cycles and showed a significant overall decrease (median 52.4%, IQR 33.3–64.9%; Wilcoxon *p* < 0.001). Among PES cycles with both serological and clinical data (*n* = 7), changes in AChR antibody titers showed no significant association with MG-ADL improvement (Pearson r = −0.12, *p* = 0.79; Spearman *ρ* = −0.34, *p* = 0.46). Figure 4 illustrates individual trajectories of total IgG (Figure 4A) and AChR antibody levels (Figure 4B) across treatment cycles, respectively.

### Adjunctive therapy and real-world use

During follow-up, four patients (4/13; 31%) required temporary adjunctive therapy for episodes of clinical worsening managed outside the setting of myasthenic crisis. IVIG was administered on six occasions in four patients, and PLEX was performed twice in two patients, both of whom had previously received IVIG. These interventions were most commonly initiated in the context of intercurrent infections or prolonged intervals between efgartigimod cycles. In most cases, adjunctive therapy was associated with clinical stabilization. One patient with a highly treatment-refractory disease course required multiple adjunctive courses (two IVIG cycles and one PLEX) and subsequently discontinued efgartigimod because of lack of sustained overall clinical benefit.

### Safety and tolerability

Efgartigimod was overall well tolerated, with no treatment-related serious adverse events or infusion reactions. Mild to moderate infections occurred in 13 of 52 treatment cycles (25%), most commonly upper respiratory tract infections (*n* = 6) and lower respiratory tract infections (LRTI) (*n* = 3). In one case, a cycle was discontinued after the second infusion due to an LRTI requiring antibiotic therapy. One patient with chronic suprapubic catheter experienced recurrent urinary tract infections during two separate cycles, while another developed herpes zoster ophthalmicus after the third infusion of a cycle, resulting in prolonged antiviral therapy and postponement of the final dose. Other reported adverse effects included headache, myalgia, chills, and diarrhea in one patient. Overall, 6 cycles were postponed and one was stopped early due to intercurrent illness. All affected patients recovered fully, and none required hospitalization or permanent treatment discontinuation.

## Discussion

In this two-center retrospective real-world study from Greece and Cyprus, we describe how efgartigimod is used and managed over time in patients with AChR antibody–positive gMG in routine clinical practice at our centers, with emphasis on treatment patterns, retreatment practices, management of non-crisis clinical worsening, and adjunctive therapy use.

Overall, most patients experienced functional improvement during efgartigimod treatment cycles, with MG-ADL scores typically decreasing by ≥2 points during treatment and varying between cycles. This temporal pattern is consistent with findings from the pivotal ADAPT and ADAPT+ trials and from multiple real-world cohorts in Europe, Asia, and North America, where treatment effects are generally cycle-dependent (10–15, 17–22). Three patients (30% of the PES) had MG-ADL scores that were higher at the most recent assessment compared with their initial pre-treatment baseline, with an increase of 2 points in each case. Importantly, all three patients had previously achieved ≥2-point reductions in MG-ADL during earlier treatment cycles. Notably, only one of these patients discontinued efgartigimod because of loss of sustained benefit. In the remaining two patients, treatment was continued at the end of follow-up, reflecting individualized clinical judgment that continued therapy provided disease control compared with prior treatment, in the context of acceptable tolerability. These findings reflect, in part, the fluctuating nature of gMG in routine clinical practice and underscore the limitations of relying on a single end-of-observation assessment to capture treatment effects that vary across cycles.

In contrast to the fixed retreatment schedules used in clinical trials, inter-cycle intervals in this study were highly variable and were determined by clinician judgment based on symptom recurrence, comorbidities, and practical considerations. Such individualized retreatment practices are increasingly recognized in real-world reports (13, 17, 23, 24) and provide important context for interpreting both transient improvements and late clinical fluctuations during follow-up.

Most patients in this cohort were treated for refractory disease or symptom relapse outside the setting of myasthenic crisis, despite corticosteroids and, in a subset, concomitant conventional immunosuppressive therapy or maintenance IVIG. Treatment decisions were individualized and based on patient-specific clinical considerations rather than a uniform treatment algorithm. In two immunosuppressant-naïve patients with long-standing gMG, residual limb weakness, and intermittent symptomatic fluctuations, efgartigimod was selected in preference to initiating non-selective immunosuppressive therapy. Although reversal of fixed weakness is not expected with FcRn blockade, treatment was chosen in the presence of ongoing fluctuating disease activity and the targeted mechanism and tolerability profile of efgartigimod (3, 4, 6, 8, 10, 11). In three patients with a history of extrathymic malignancy, efgartigimod was preferentially selected to minimize systemic immunosuppression in settings where conventional agents may warrant increased oncologic vigilance. While no safety concerns emerged during follow-up, longer observation periods are required to better define long-term safety in this population (4, 9–11). In addition, one patient initiated efgartigimod after secondary loss of response to ravulizumab, illustrating real-world sequential use of FcRn inhibition following complement blockade. Clinical evidence to guide such sequencing strategies remains limited, highlighting the need for further comparative and longitudinal studies (9, 17, 25, 26).

Efgartigimod was also used in selected patients during periods of MSE. In one patient, treatment was initiated with the primary aim of facilitating corticosteroid dose reduction prior to planned thymectomy, in the context of prolonged high-dose corticosteroid exposure and concern for peri-operative complications. The remaining two patients had late-onset gMG and received efgartigimod as bridging therapy while awaiting the onset of action of a conventional non-steroidal immunosuppressive agent. In one case, this approach followed severe exacerbations requiring rescue therapy in the months prior to efgartigimod initiation, whereas in the other long-term oral corticosteroid therapy was contraindicated because of severe osteoporosis.

Across the cohort, corticosteroid tapering was achieved in the majority of patients during follow-up, with an overall reduction of approximately 50% from baseline doses. While corticosteroid sparing represented the primary treatment objective in one patient, in others it emerged as a secondary outcome during treatment. These observations are in line with real-world data from Europe and the United States, where 50–70% of patients have been able to reduce or discontinue corticosteroids following repeated efgartigimod cycles (13, 27). In routine clinical practice, this effect may be particularly relevant for patients requiring long-term immunosuppression, for whom minimizing cumulative corticosteroid exposure remains an important therapeutic goal (3).

During follow-up, two patients permanently discontinued efgartigimod: one due to gradual attenuation of treatment effect over successive treatment cycles, and one following a new diagnosis of malignancy deemed unrelated to therapy.

No patients in this cohort experienced myasthenic crisis during the observation period. At our centers, management of myasthenic crisis follows established consensus guidelines, with IVIG or PLEX used as first-line therapies for acute deterioration requiring rapid clinical stabilization (3). Episodes of clinical worsening observed during efgartigimod therapy occurred outside the context of myasthenic crisis and did not involve respiratory failure requiring airway protection, or the need for intensive care support. Although IVIG and PLEX are cornerstone therapies for the management of myasthenic crisis (3), in our routine clinical practice they are also used for severe non-crisis exacerbations when there is concern for further progression. In our cohort, adjunctive IVIG and/or PLEX was administered in four patients during the observation period in this preventive context to stabilize disease activity and avert further deterioration, after which efgartigimod could be resumed as part of longitudinal management. Such episodes suggest that, in routine clinical practice, transient fluctuations in disease control may reflect intercurrent illness, changes in concomitant medications, or treatment spacing rather than loss of drug efficacy. Recent retrospective studies have explored the use of efgartigimod in selected patients with impending myasthenic crisis; however, the available evidence remains limited and does not establish an alternative to standard rescue therapies (28). Further prospective investigation is required to clarify its role in this setting, particularly given the non-immediate onset of clinical effect associated with FcRn blockade (10).

Efgartigimod was well tolerated, with no serious treatment-related adverse events or hospitalizations. Mild infections, mostly upper or lower respiratory, occurred in a few patients, and 1 cycle was stopped early due to pneumonia. A single case of herpes zoster ophthalmicus led to postponement of the final infusion but resolved without sequelae. Several patients were receiving concomitant immunosuppressive therapy including oral corticosteroids, which may have contributed to infection susceptibility. Occasional non-infectious reactions such as headache, chills, myalgia, or diarrhea were mild and self-limited. Overall, all affected patients recovered fully, and no permanent treatment discontinuations occurred due to side effects.

In line with the established pharmacodynamic profile of FcRn blockade, total IgG levels declined substantially and consistently following each efgartigimod cycle, with rapid recovery during inter-cycle intervals (7, 8). The magnitude of IgG reduction observed in our cohort (median 56.7%) closely mirrors that reported in pivotal trials and real-world studies, confirming effective target engagement in clinical practice (10, 11, 13, 14, 29). However, within the PES, no significant correlation was observed between the extent of IgG or AChR antibody reduction and clinical improvement. This finding suggests that the degree of antibody lowering alone does not directly determine the magnitude of functional benefit, consistent with previous observations from controlled and real-world cohorts (10–13, 17), and indicating that additional immunoregulatory mechanisms may contribute to clinical improvement (30, 31).

### Strengths and limitations

This study provides the first real-world analysis of efgartigimod use in Greece and Cyprus, drawing on detailed longitudinal data from two national neuromuscular referral centers. The availability of cycle-level MG-ADL assessments, corticosteroid dosing, and serological measurements enabled an integrated evaluation of clinical, pharmacodynamic, and treatment-pattern outcomes over time. Importantly, the pragmatic design—with individualized retreatment intervals, concomitant immunosuppressive use, and real-world management of clinical worsening—closely reflects routine clinical practice and enhances the clinical relevance of the findings.

Several limitations should be acknowledged. The retrospective, observational, and uncontrolled design, together with the small sample size, limits statistical power and generalizability. Only patients within the PES could be meaningfully assessed for MG-ADL improvement, as those treated during periods of MSE were analyzed separately for maintenance or steroid-sparing purposes. Serological data were not available for all treatment cycles, and background immunosuppressive regimens—including corticosteroid dosing—were heterogeneous and could change over time. MG-ADL was the sole clinical outcome measure; the absence of complementary quantitative assessments such as QMG or MG-Composite may have limited sensitivity to subtle changes in strength or endurance. In addition, sustained response duration as defined in ADAPT could not be systematically evaluated due to variable follow-up timing inherent to real-world practice. Differences in follow-up duration and retreatment intervals may also have influenced comparisons across cycles. Moreover, access to efgartigimod in Greece and Cyprus is subject to national reimbursement and approval procedures, which may have contributed to delays in treatment initiation and variability in retreatment timing. Despite these limitations, the study provides clinically meaningful insight into how FcRn blockade is applied and managed in everyday practice across diverse clinical scenarios.

## Conclusion

In this two-center retrospective real-world study, we describe the use and longitudinal management of efgartigimod in patients with AChR antibody–positive generalized myasthenia gravis in routine clinical practice. Most patients experienced repeated functional improvement during treatment cycles, although the magnitude and durability of response varied across individuals.

Efgartigimod was integrated into care across a range of real-world clinical scenarios, including refractory disease, non-crisis symptom relapse, corticosteroid tapering, maintenance of minimal-symptom expression, and bridging before thymectomy or initiation of conventional immunosuppression. Episodes of clinically significant worsening occurred outside the setting of myasthenic crisis and were managed with established rescue therapies, after which efgartigimod could often be resumed as part of ongoing disease control.

Variability in clinical status over time, including higher MG-ADL scores at later assessments in some patients despite earlier improvement, underscores the fluctuating nature of generalized myasthenia gravis and the limitations of relying on single end-of-observation evaluations in real-world settings. Overall, these findings illustrate how FcRn blockade is applied pragmatically as part of individualized, longitudinal disease management, complementing established immunomodulatory therapies. Further prospective studies in larger cohorts are warranted to better define optimal retreatment strategies, predictors of sustained benefit, and the positioning of efgartigimod across diverse real-world clinical contexts, including its potential role in impending myasthenic crisis.

## Data Availability

The raw data supporting the conclusions of this article will be made available by the authors, without undue reservation.
