# Supplementary material for: Real-world use of efgartigimod in acetylcholine receptor antibody–positive generalized myasthenia gravis: experience from two centers in Greece and Cyprus
Source: Front Neurol. 2026 Jan 21;17:1755374. doi: 10.3389/fneur.2026.1755374 (PMC12867891; doi:10.3389/fneur.2026.1755374)
Supplement: Supplementary file 1 [file Table_1.docx]

**Supplementary Table 1. Clinical characteristics and treatment summary of patients with AChR-positive generalized myasthenia gravis treated with efgartigimod.** Disease duration refers to the time from symptom onset to initiation of efgartigimod. MGFA classification = Myasthenia Gravis Foundation of America clinical classification at treatment baseline. MG-ADL = Myasthenia Gravis Activities of Daily Living scale; baseline and latest values correspond to the first and most recent efgartigimod cycle, respectively. ΔMG-ADL per cycle = mean change in MG-ADL score per cycle (negative values indicate improvement). Inter-cycle interval = mean time between consecutive efgartigimod cycles. Prednisone-equivalent dose (mg) before the first and at the last recorded cycle. For alternate-day regimens, the mean daily equivalent dose was calculated (e.g., 20/10 mg → 15 mg/day). Baseline IS = concomitant oral immunosuppressant(s) at treatment initiation. Rescue therapies include intravenous immunoglobulins (IVIG) and/or plasma exchange (PLEX) administered during efgartigimod treatment. MSE = minimal-symptom expression (MG-ADL 0–1); denoting patients with minimal baseline symptoms in whom MGFA classification was not applicable. AZA = azathioprine; MMF = mycophenolate mofetil.

| Patient | Disease duration (years) | MGFA classification (baseline) | Baseline MG-ADL | Latest MG-ADL | Mean ΔMG-ADL per cycle | Cycles (n) | Inter-cycle interval (weeks) | Prednisone-equivalent dose (mg, pre→last) | Baseline IS | Reasons for efgartigimod initiation | Rescue therapies during treatment | Comments |
| --- | --- | --- | --- | --- | --- | --- | --- | --- | --- | --- | --- | --- |
| P1 | 21.5 | IIIb | 9 | 3 | −4.00 | 6 | 9.8 | 15 → 10 | MMF | Refractory disease | IVIG (3×) | — |
| P2 | 10.6 | IIIa | 7 | 0 | −7.00 | 1 | (single) | 25 → 15 | None | Non-crisis symptomatic relapse | — | — |
| P3 | 1.3 | IVb | 9 | 5 | −6.25 | 4 | 8.7 | 10 → 0 | MMF | Refractory disease | IVIG (1×), PLEX (1×) | Severe dysphagia, NG-tube feeding. MMF discontinued due to lymphopenia |
| P4 | 17.3 | IIIa | 10 | 12 | −2.38 | 8 | 16.6 | 15 → 15 | MMF | Refractory disease | IVIG (2×), PLEX (1×) | Discontinued due to loss of effect |
| P5 | 1.1 | IIIb | 8 | 6 | −4.00 | 5 | 26.8 | 25 → 12.5 | AZA | Refractory disease, corticosteroid-sparing | — | AZA discontinued due to lymphopenia |
| P6 | 8.2 | IIIb | 3 | 5 | −1.50 | 6 | 11.6 | 25 → 10 | None | Refractory disease, corticosteroid-sparing | IVIG (1×) | Extrathymic malignancy |
| P7 | 0.2 | (MSE) | 1 | 0 | −0.33 | 3 | 16.0 | 10 → 8.75 | None | Maintenance | — | Extrathymic malignancy |
| P8 | 6.4 | IIIa | 6 | 3 | −2.00 | 4 | 9.0 | 10 → 5 | None | Refractory disease | — | Extrathymic malignancy; discontinued due to new malignancy |
| P9 | 1.2 | (MSE) | 0 | 0 | 0.00 | 1 | (single) | 10 → 7.5 | None | Bridging to conventional immunosuppression | — | — |
| P10 | 0.8 | (MSE) | 0 | 0 | 0.00 | 4 | 10.3 | 55 → 20 | None | Bridging before thymectomy, corticosteroid-sparing | — | — |
| P11 | 9.4 | IIIa | 4 | 1 | −2.00 | 3 | 10.0 | None | None | Residual weakness with symptom fluctuation | — | Immunosuppressant-naïve |
| P12 | 4.6 | IIIb | 3 | 5 | −0.60 | 5 | 7.3 | 15 → 15 | None | Refractory disease, switch from ravulizumab | — | — |
| P13 | 31.5 | IIIa | 7 | 3 | −1.50 | 2 | 14.0 | None | None | Residual weakness with symptom fluctuation | — | Immunosuppressant-naïve |
